# Melatonin in Alzheimer's disease and other neurodegenerative disorders

**DOI:** 10.1186/1744-9081-2-15

**Published:** 2006-05-04

**Authors:** V Srinivasan, SR Pandi-Perumal, DP Cardinali, B Poeggeler, R Hardeland

**Affiliations:** 1 Department of Physiology, School of Medical Sciences, University Sains Malaysia, Kampus Kesihatan, 16150, Kubang kerian, Kelantan, Malaysia; 2 Comprehensive Center for Sleep Medicine, Division of Pulmonary, Critical Care and Sleep Medicine, Mount Sinai School of Medicine, 1176 – 5^th ^Avenue, New York, NY 10029, USA; 3 Departamento de Fisiología, Facultad de Medicina, Universidad de Buenos Aires, 1121, Buenos Aires, Argentina; 4 Johann Friedrich Blumenbach Institute of Zoology and Anthropology, University of Goettingen, Berliner Str. 28, D-37073 Goettingen, Germany

## Abstract

Increased oxidative stress and mitochondrial dysfunction have been identified as common pathophysiological phenomena associated with neurodegenerative disorders such as Alzheimer's disease (AD), Parkinson's disease (PD) and Huntington's disease (HD). As the age-related decline in the production of melatonin may contribute to increased levels of oxidative stress in the elderly, the role of this neuroprotective agent is attracting increasing attention. Melatonin has multiple actions as a regulator of antioxidant and prooxidant enzymes, radical scavenger and antagonist of mitochondrial radical formation. The ability of melatonin and its kynuramine metabolites to interact directly with the electron transport chain by increasing the electron flow and reducing electron leakage are unique features by which melatonin is able to increase the survival of neurons under enhanced oxidative stress. Moreover, antifibrillogenic actions have been demonstrated *in vitro*, also in the presence of profibrillogenic apoE4 or apoE3, and *in vivo*, in a transgenic mouse model. Amyloid-β toxicity is antagonized by melatonin and one of its kynuramine metabolites. Cytoskeletal disorganization and protein hyperphosphorylation, as induced in several cell-line models, have been attenuated by melatonin, effects comprising stress kinase downregulation and extending to neurotrophin expression. Various experimental models of AD, PD and HD indicate the usefulness of melatonin in antagonizing disease progression and/or mitigating some of the symptoms. Melatonin secretion has been found to be altered in AD and PD. Attempts to compensate for age- and disease-dependent melatonin deficiency have shown that administration of this compound can improve sleep efficiency in AD and PD and, to some extent, cognitive function in AD patients. Exogenous melatonin has also been reported to alleviate behavioral symptoms such as sundowning. Taken together, these findings suggest that melatonin, its analogues and kynuric metabolites may have potential value in prevention and treatment of AD and other neurodegenerative disorders.

## Introduction

Oxidative damage has been suggested to be the primary cause of aging and age-associated neurodegenerative diseases like Alzheimer's disease (AD), Parkinson's disease (PD), and Huntington's disease (HD). This concept is based on the free radical hypothesis of aging as proposed by Harman [1]. Many reviews on AD present compelling evidence for a decisive participation of severe oxidative stress in the development of neuropathology seen in this disease [2-9]. Immunohistochemical proof that enhanced oxidative stress and damage to biomolecules are hallmarks of the disease and its progression was first presented by Pappolla et al. [3]. This study confirmed findings demonstrating increased levels of lipid peroxidation *in vitro *observed in autopsy samples of brains afflicted by AD [10]. Because of its high rate of oxygen consumption and its high content of polyunsaturated fatty acids, the brain exhibits increased vulnerability to oxidative stress. Elevated lipid peroxidation, as found in the brains of AD patients, not only reveals oxidative stress [10-13], but also exerts secondary effects on protein modification, oxidation and conformation [14,15]. Increased protein and DNA oxidation also occurs in AD. Measurements of protein carbonyl, 3,3'-dityrosine and 3-nitrotyrosine in *post mortem *brain samples from AD patients have shown increased oxidative and nitrosative protein modification in the hippocampal and neocortical regions, but not in the cerebellum [14,16-18]. Free radical attack on DNA results in strand breaks, DNA-protein cross linkage, and base modification. Double- and single-strand breaks were elevated in AD cortex and hippocampus, but this has to be largely attributed to apoptotic fragmentation [19,20]. Enhanced oxidative DNA modification is, however, also demonstrable, mostly as 8-hydroxy-2'-deoxyguanosine (8-OHdG) [21-23], a product primarily formed by attack of hydroxyl radicals [24], but other modified bases such as 8-OH-adenine have also been demonstrated [25]. Augmented free radical damage to lipids, proteins and nucleic acids has been reported for the substantia nigra of parkinsonian patients [26]. Therefore, numerous compounds with antioxidant properties have been suggested for treatment of AD and other neurodegenerative diseases [27-30]. Among these substances, melatonin is unique for several reasons: it is a natural compound synthesized in the pineal gland and other body tissues; it can be released by the pineal gland via the pineal recess into the cerebrospinal fluid (CSF), in much higher concentrations than into the circulation [31,32]; its production decreases with the advancement of age, a fact which has been suggested to be one of the major causes of age-associated neurodegenerative diseases [8,9,33,34]. This review focuses on the role of melatonin in the etiology of AD and other neurodegenerative disorders and on the therapeutic potential of melatonin in these pathologies, including effects on sleep and behavior.

### Melatonin: sources, dynamics and signaling

Melatonin is a methoxyindole secreted mainly, but not exclusively by the pineal gland. Once formed melatonin is not stored within the pineal gland but diffuses out into the capillary blood [35] and CSF [31]. Melatonin arrives early in the CSF of the third ventricle as compared to that of the lateral ventricles. Levels of melatonin released to the CSF were found to be 5 to 10 (up to 30) times higher than those simultaneously measured in the blood [31], whereas spinal CSF values did not much deviate from those in the serum. These findings indicate uptake of melatonin by the brain tissue, perhaps also metabolization to other compounds, such as substituted kynuramines, which are thought to display protective properties. Brain tissue may have higher melatonin levels than other tissues in the body [32].

It must be noted that the levels of a relatively lipophilic substance like melatonin reaching neurons under physiological or pharmacological conditions can differ considerably from circulating hormone concentrations. In early studies using high-pressure liquid chromatography [36] or radioimmunoassay [37,38], hypothalamic melatonin concentrations were found to be about 50 times greater than in plasma. Two compartments of melatonin have been proposed to exist, which differentially affect physiological functions: in the plasma, melatonin would mainly act on peripheral organs, whereas, in the CSF, it might affect neurally mediated functions at a much higher concentration. Evidence interpreted as supporting this view has been presented in a study demonstrating that melatonin levels in the CSF of the third ventricle were 20-fold higher than nocturnal plasma concentrations [39]. Therefore, it seems necessary to distinguish strictly between melatonin concentrations in the circulation and in tissues [40,41].

While tissue melatonin sometimes shows only moderate circadian amplitudes [40-42], circulating melatonin exhibits one of the most pronounced circadian rhythms known, at least prior to aging. The peak concentration occurs at night and is higher in younger age (18–54 yrs). With some exceptions [43,44], a strong decline of melatonin during aging has been consistently reported by many investigators [45-51].

The age-associated decline in melatonin production and the flattening of the melatonin rhythm may be major contributing factors to the increased levels of oxidative stress and associated degenerative changes seen at old age. However, individuals of the same chronological age can exhibit considerable deviations in the degree of senescence-associated functional impairment. Some discrepancies between findings of different investigators can be attributed to the interindividual variation in melatonin levels of the same age group [8].

It is the physiological age of an individual rather than the chronological age that determines one's melatonin production. The varying extent of degenerative changes of cells and tissues may correspond to differences of melatonin production in the body [8].

Melatonin is involved in the control of various physiological functions such as coordination of other circadian rhythms including that of the central pacemaker, the suprachiasmatic nucleus (SCN) [52-55], sleep regulation [56,57], immune function [58,59], growth inhibition of malignant cells [60], blood pressure regulation [61,62], retinal functions [63-65], modulation of mood and behavior [66-68], free radical scavenging and other antioxidant actions [40,41,69-71]. Many effects of melatonin, especially those concerning the circadian pacemaker system, are mediated by the G_i_-protein (alternately G_0 _or G_q_) coupled membrane receptors MT_1 _and MT_2 _[72-74]. Additional binding sites exist. A previously assumed membrane receptor MT_3 _was shown to represent an enzyme, quinone reductase 2 [75], which may participate in antioxidative protection through elimination of prooxidant quinones [40,76].

Other effects may be related to nuclear receptors of lower ligand sensitivity, RORα, which exists in at least four variants, and RZRβ [77,78], but in these cases functional significance and target genes are less clear. Effects on the immune system have been partially attributed to these nuclear binding sites, but membrane receptors are obviously also involved [55,59]. To which extent the upregulation of antioxidant enzymes depends on nuclear receptors deserves clarification in detail. γ-Glutamylcysteine synthase, the rate-limiting enzyme of glutathione biosynthesis, is stimulated by melatonin at the transcriptional level; EMSA (electrophoretic mobility shift analyses) data have shown melatonin-dependent rises in DNA binding not only of AP-1, but also of RZRβ/RORα [79].

Further effects of melatonin do not require any of these membrane and nuclear receptors, since the indoleamine is able to directly bind to calmodulin [80], thereby inhibiting CaM-kinase II, and, moreover, to cause activation and Ca^2+^-dependent membrane translocation of protein kinase C [81], at concentrations in the nanomolar range, at a near-physiological level. Since these effects are related to the long-known cytoskeletal changes induced by melatonin, they may become of interest with regard to the abnormalities in cytoskeletal architecture and phosphorylation of cytoskeleton-associated proteins in AD. This aspect may be of higher relevance than obvious at first glance. Since cytoskeletal alterations, as observed in AD as well as tau hyperphosphorylation and related upregulations in the MAP kinase pathway, can be mimicked by the protein phosphatase inhibitor okadaic acid [82-85], the counteraction by melatonin of okadaic acid-induced AD-like lesions seems to indicate a common level of action [86,87]. Interestingly, okadaic acid also caused oxidative stress, which was again antagonized by melatonin [88], in an MT_1_-receptor-independent fashion [89].

The relationship between melatonin and Ca^2+ ^and, thus, with the activity state of the cell may be more profound than previously thought. The indoleamine was also found to bind, with a physiologically relevant K_d _of about 1 nM, to calreticulin, a Ca^2+^-binding protein not only present in the endoplasmic reticulum, but also in the nucleus [90]. These findings may turn out to be relevant with regard to the involvement of Ca^2+ ^overload in overexcited neurons and cell death in neurodegenerative processes.

Concerning the antioxidative protection against amyloid-β, agonists of the MT_1 _and MT_2 _membrane receptors without antioxidant properties were not effective in neuroblastoma cells and primary hippocampal neurons, so that the neuroprotective and antiamyloidogenic properties of melatonin appeared to be independent of these receptors [91].

### Melatonin and Alzheimer's disease

AD is an age-associated neurodegenerative disease that is characterized by a progressive loss of cognitive function, loss of memory, and other neurobehavioral manifestations. In spite of a large number of studies undertaken, the etiology of AD is largely unknown. Many mechanisms have been proposed, including genetic predispositions (e.g., expression levels and subforms of presenilins and ApoE), inflammatory processes associated with cytokine release, oxidative stress, and neurotoxicity by metal ions [92-99]. Pathological manifestations of AD include extracellular plaques of β-amyloid and intracellular neurofibrillary tangles composed of abnormally bundled cytoskeletal fibers. The deposition of amyloid plaques is thought to destabilize neurons by mechanisms which require further clarification. Tangles are associated with hyperphosphorylation of tau, a microtubule-associated protein, and of neurofilament H/M subunits, processes that lead to misfolding and accumulation of these proteins, along with a disruption of microtubules [94,98,100-103]. With regard to oxidative stress, prooxidant properties of the free amyloid-β molecule (Aβ) may be decisive, which is Fenton-reactive due to bound copper, and can, therefore, lead to hydroxyl radical-induced cell death. Additionally, Aβ initiates flavoenzyme-dependent rises in intracellular H_2_O_2 _and lipid peroxides, which also cause radical generation [27,104,105]. Rises in Aβ protein have, in fact, been shown to induce oxidative stress [106]. Moreover, an impairment of neurotrophin activity on associated tyrosine kinase receptors has been suggested to represent an important factor in AD pathology [107-110].

With regard to the involvement of oxidative stress in AD, melatonin represents an interesting agent, since it displays multiple properties by which oxidative stress is antagonized [33,40,41]. In addition, other actions of melatonin exceed this aspect, but seem to have a beneficial potential in AD, too, as will be discussed in detail.

Accumulation of aggregated Aβ and tau hyperphosphorylation are highly common phenomena observed during aging of primates and other mammals [111]. Though Aβ contributes directly or indirectly to neuronal degeneration, the potential of amyloid to cause AD depends on the individual's susceptibility to Aβ-mediated toxicity [28]. In AD brains, oxidative end products are found to be significantly elevated. Metabolites of lipid peroxidation and oxidatively modified proteins and DNA are abundantly present in post mortem brain samples of AD patients [5,10,112,113]. Inflammatory reactions associated with microglia and generation of nitric oxide (NO)-derived radicals contribute to cell stress and seem to be important especially in the degeneration of proximal neurons [114]. Nevertheless, the inflammatory component in AD is clearly different from normal inflammation, since some classical hallmarks such as neutrophil infiltration and edema are usually absent, whereas other characteristics including acute-phase proteins and cytokines can be identified [17].

It seems important to distinguish between the extra- and intracellular sources of oxidants. Extracellular attack of neurons by oxidants may result either from inflammatory responses or from free radicals formed by Fenton-reactive Aβ molecules [27]. Intracellular oxidative stress seems to be indirectly caused by Aβ, effects that may involve receptors or other surface molecules able to transduce oxidotoxicity [115-118]. Recently AD has been related to mitochondrial dysfunction [87,119]. This conclusion is based on several lines of evidence. First, cells depleted of mitochondrial DNA become insensitive to Aβ toxicity [120]. Second, cybrid cells (cytoplasmic hybrid cells: mitochondrial DNA-depleted recipients of mitochondria from other sources) containing mitochondria from AD patients have shown enhanced vulnerability to Aβ [121]. Third, cybrids with mitochondria from sporadic AD, which is associated with lower cytochrome c oxidase activity, due to a defective gene [122-126], exhibit various other signs of mitochondrial dysfunction, such as disturbed Ca^2+ ^homeostasis [122] and Na^+^/Ca^2+ ^exchange [127], enhanced formation of reactive oxygen species [122,128,129], lowered mitochondrial membrane potential [130] and, sometimes, abnormal morphology [125,131].

These abnormalities may now be seen under aspects reminiscent of other mitochondrial diseases associated with pathological oxidant formation [cf. ref. [132]], since similar changes are also present in PD, HD and Friedreich's ataxia, and sometimes already demonstrated in respective cybrid models [124,128,131]. Collectively, all evidence convincingly demonstrates that the neural tissue of AD patients is subjected to increased oxidative stress. Therefore, its attenuation or prevention should be the goal of a strategic treatment of this neurodegenerative disease. However, a simplistic concept aiming to reduce oxidative damage and its consequences by applying classical radical scavengers is obviously insufficient. Vitamins E and C have been used for the treatment of AD patients with only limited success. Although several studies demonstrated a reduction in lipid peroxidation [133,134], epidemiological data showed only minor or no clear-cut effects [135-138]. Moreover, these compounds remained relatively inefficient in preventing Aβ toxicity and fibrillogenesis [139-141].

In this regard, melatonin and other structurally related indolic compounds, such as indole-3-propionic acid, proved to be more potent [28,140,142-144]. This may not only be a matter of radical-scavenging capacity, but also involve additional effects of these compounds and, perhaps, their metabolites. In particular, antifibrillogenic effects were observed *in vitro *[140,144], but also *in vivo *in transgenic mouse models [145,146]. Moreover, protection from Aβ toxicity was observed, especially at the mitochondrial level [91,143]. For these reasons, melatonin appears as an antioxidant of superior potency, with additional effects relevant to intervention in AD.

### Multiple antioxidant actions of melatonin in the brain: implications for neuroprotection

As pointed out, antioxidative protection is not limited to radical scavenging and must be seen in a broader context involving many different mechanisms. Melatonin exerts several actions which collectively contribute to the prevention of oxidative damage assuring survival of cells even under adverse conditions. We shall, therefore, analyze in detail the different sources of oxidative stress and damage which allow for a broad spectrum of different counteractions by melatonin and a specific response to this antioxidant and adaptogenic agent. It should be kept in mind that coincidence does not indicate causality: Oxidative damage may sometimes be the consequence rather than the cause of the pathology observed in neurodegenerative disorders, with the mechanisms of protection exerted by melatonin often being quite complex and even interdependent. Neuroprotective effects induced by melatonin may act in concert to reduce oxidative stress and damage. Since it is not easily possible to distinguish between direct and indirect antioxidant actions mediated by melatonin, it is of utmost importance not to arrive at preliminary conclusions, which do not reflect the complexity of the multiple responses to this highly potent neuroprotective agent.

For several reasons, the central nervous system (CNS) exhibits a relatively high susceptibility to oxidative stress. As mentioned, one of these is a high oxygen consumption rate that inevitably accounts for increased generation of free radicals. Moreover, the brain is relatively rich in polyunsaturated fatty acids, a property which is not unfavorable *per se*, but which can become problematic under oxidative stress; especially docosahexaenoic acid is easily peroxidized, and this process has been discussed in relation to neurodegenerative diseases including AD [5,147-149]. Lipid peroxidation was found to initiate secondarily oxidative protein modifications, particularly in the AD brain [14]. Numerous publications have demonstrated that lipid peroxidation can be suppressed by melatonin, and much of this work has been carried out in the CNS [33,71,150]. Fewer data are available with direct relevance to AD. Melatonin did not only antagonize tau hyperphosphorylation induced by the PI3 kinase inhibitor wortmannin, but also a wortmannin-dependent stimulation of lipid peroxidation [151]. Again, such findings shed light on the complexity of actions. While suppression of lipid peroxidation may be seen, at first glance, solely as an effect of an antioxidant, perhaps only by radical scavenging, the relationship to altered protein kinase activities reveals the involvement of additional actions in the metabolism.

Another aspect of vulnerability of the CNS is related to the availability of other, enzymatic and low molecular weight antioxidants. Antioxidant enzymes usually attain only moderate activities in the brain, but are in any case not that low as sometimes incorrectly stated (especially with regard to catalase). Among low molecular weight antioxidants, glutathione levels are comparable to those of other tissues, but ascorbate is usually by one order of magnitude higher than in the circulation. This basically protective scavenger turns into an extremely unfavorable and prooxidant agent in the presence of elevated iron concentrations, since the reductant is driving a Fenton reaction-based redox cycling. Iron levels are high in certain brain areas and, in addition, damage to the brain tissue, ischemia or neurotrauma can further mobilize iron so that radical-dependent destruction of biomolecules and cell death are strongly enhanced [152]. Counteractions by melatonin against damage by Fenton reagents have been repeatedly demonstrated [153-155].

These effects are related to the remarkable efficacy of melatonin to scavenge various free-radicals, in particular, the extremely reactive hydroxyl radical [69,156-159]. This property, which has been repeatedly reviewed [33,40,41,160-162], extends also to carbonate radicals (CO_3_•^-^) [163], reactive nitrogen species and to actions of metabolites of melatonin, such as cyclic 3-hydroxymelatonin, *N*^1^-acetyl-*N*^2^-formyl-5-methoxykynuramine (AFMK) and *N*^1^-acetyl-5-methoxykynuramine (AMK) [40,164-168]. Carbonate radicals, which have been shown to interact with both melatonin and AMK, abstract electrons (melatonyl cation radicals formed by CO_3_•^- ^were demonstrated) or alternately, hydrogen atoms. •NO exhibits nitrosation reactions with melatonin, AFMK and AMK, whereas peroxynitrite-derived radicals, such as •NO_2 _and •OH (from ONOOH) or •NO_2 _and CO_3_•^- ^(from ONOOCO_2 _^-^) lead to nitration of these molecules.

Another poorly understood, but possibly important field is that of interactions with other antioxidants. In both chemical and cell-free systems, melatonin was shown to potentiate the effects of ascorbate, Trolox (a tocopherol analog), reduced glutathione, or NADH, in a non-additive and synergistic manner [158,165,169,170]. These findings indicate multiple interactions, via redox-based regeneration of antioxidants transiently consumed. Also *in vivo*, under conditions of long-lasting experimental oxidative stress, melatonin was shown to prevent decreases in ascorbate and α-tocopherol levels [171]. It would be important to know whether this effect, to date only shown in the liver, may be demonstrable in the CNS, too.

Contrary to classical antioxidants, melatonin exerts several additional effects, which contribute either directly or indirectly to the decrease of free radicals, and some of these actions are particularly relevant to or specific for the brain. Antioxidant enzymes were repeatedly shown to be upregulated by melatonin. While activities or gene expression of enzymes like Cu,Zn- and Mn-superoxide dismutases and hemoperoxidase/catalase were stimulated by melatonin in a highly variable, tissue-specific fashion and usually only moderately in the CNS [40,41], glutathione peroxidase was consistently and considerably upregulated in the brain [40,41,160,172,173]. Glutathione reductase was usually found to rise after glutathione peroxidase, perhaps reflecting a secondary control by GSSG [40,174-177][178]. Additional stimulations of glucose-6-phosphate dehydrogenase [176] and γ-glutamylcysteine synthase [79,161,177] indirectly support the action of glutathione peroxidase by providing reducing equivalents (NADPH) for the action of glutathione reductase and by increasing the rate of glutathione synthesis, respectively. In addition, melatonin downregulates prooxidant enzymes such as lipoxygenases [161,177] and NO synthases [40,41,76,161,176,177,179-185]. In this way, oxidative and nitrosative damage is attenuated, not only by avoiding peroxynitrite-derived radicals, but also by reducing NO-dependent neuronal excitation, and by antagonizing inflammatory reactions. The antiinflammatory potential of melatonin extends to downregulation of cyclooxygenase 2, an effect which may represent an action of the metabolite AMK [40,186], a substance which is additionally a cyclooxygenase inhibitor much more potent than acetylsalicylic acid [187]. Signaling mechanisms of AMK, in terms of receptors and interactions of transcription factors with the cyclooxygenase-2 promoter, have not been investigated to date.

Especially in the brain, melatonin contributes indirectly to the avoidance of radical formation, owing to several actions which are frequently overlooked, but which may be highly relevant in practice. First, melatonin is known to exert pronounced antiexcitatory and antiexcitotoxic effects, associated with inhibition of calcium influx and NO release and, consequently, prevention of the enhanced, excitation-dependent generation of free radicals [40]. Melatonin was shown to possess strong anticonvulsant properties and to counteract efficiently the actions of various excitotoxins [188]. When analyzed in detail, neuroprotection by melatonin against excitotoxins turned out to be a superposition of antiexcitatory and direct antioxidant effects, as shown for glutamate and its agonists, ibotenate, kainic acid, domoic acid, and, in particular, also for quinolinic acid (summarized by Hardeland [40]).

Indirect antioxidative protection in terms of radical avoidance may be also assumed for the chronobiological role of melatonin as an endogenous regulator of rhythmic time structures. The importance of appropriate timing for maintaining low levels of oxidative damage has been overlooked for quite some time. However, it turned out that temporal perturbation as occurring in short-period or arrhythmic circadian clock mutants leads to enhanced oxidative damage [76]. This action may be particularly important under the aspect of melatonin supplementation in the elderly, who exhibits a strongly reduced amplitude in the circadian melatonin rhythm, and in the AD patients in which the circadian system is disturbed. Finally, radical avoidance under the influence of melatonin is also a consequence of mitochondrial effects, as exerted by the indoleamine and by its metabolite AMK.

### Safeguarding of mitochondrial electron flux and metabolism by melatonin

With regard to the mitochondrial aspect of AD and other neurodegenerative diseases – concerning radical generation, excitation-dependent calcium overload and its consequences for the mitochondrial membrane potential and for the permeability transition pore (mtPTP), involvement in apoptosis and sensitivity towards excitotoxins including Aβ- the actions of melatonin at the level of this important cellular compartment deserve particular attention.

The electron transport chain (ETC) represents a major source of reactive oxygen species (ROS) within the cell, due to electron leakage towards molecular oxygen [189]. Complexes I and III of the ETC have been identified as the two principal sites of superoxide anion (O_2_•^-^) generation [190]. While much of the O_2_•^- ^is released from complex III to either side of the inner membrane [191], the iron-sulfur cluster N2 of complex I appears to be the main site of O_2_•^- ^release to the matrix [190,192-194]. This seems to hold also for the brain, at least, under normal conditions. The fate of O_2_•^- ^can be different. A certain proportion re-donates electrons to the ETC at cytochrome c [195,196]. Another fraction is converted to H_2_O_2 _and O_2 _by the mitochondrial, manganese-containing subform of superoxide dismutase (MnSOD) [197]. However, H_2_O_2 _produced by cytosolic Cu,Zn-SOD can likewise enter mitochondria owing to its high membrane permeability. A certain amount of H_2_O_2 _is eliminated intramitochondrially by interaction with cytochrome c [195,196], while another fraction should be detoxified by peroxidases; though, the destruction of this oxidant and potential source of hydroxyl radicals is never complete. A third fraction of O_2_•^- ^combines with NO, having a similar affinity to this oxygen radical as SODs, to give peroxynitrite, a source of hydroxyl and carbonate radicals as well as NO_2_, in other words, an additional origin of destruction and nitration of proteins [198] or aromates [168,198].

Mitochondria are not only a major site of ROS generation, but also the primary target of attack for ROS and reactive nitrogen species (RNS) [199]. Damage to the mitochondrial respiratory chain can either cause breakdown of the proton potential, opening of the mtPTP and, thus, induce apoptosis or lead to further generation of free radicals maintaining a vicious cycle, which ultimately also ends up in cell death [189], either of the necrotic or apoptotic type [200].

Findings of several investigators indicate that the neuroprotective role of melatonin in AD and PD is primarily due to mitochondrial effects. This is not only a matter of radical scavenging (see above) – which may support protection, but can be only of limited efficacy for reasons of stoichiometry – but also of additional actions exceeding the direct elimination of free radicals. Some of these are rather conventional, concerning protection of mitochondrial membranes and DNA from oxidative insults, stimulation of glutathione (GSH) synthesis and support of the reduction of oxidized glutathione (GSSG) [reviews: refs. [40,41]]. Some others may be also regarded as indirect antioxidant effects of melatonin, which are, however, based on the maintenance of mitochondrial electron flux, something that is notably observed even under adverse conditions [201-206].

Melatonin's mitochondrial actions are taking place within the organelle. This statement is important since it strongly contrasts with many other antioxidants. Melatonin, disposing of a balanced amphiphilicity, crosses the cell membranes with ease and may be able to concentrate within subcellular compartments [207]. Mitochondrial accumulation has been discussed [205], but this issue has not yet been finally settled. Its amphiphilicity may allow melatonin to act at or even within the membrane. Whether effects on the fluidity of the mitochondrial inner membrane [208] reflect such a property is uncertain, since these experiments were performed under oxidative stress, which leads to membrane rigidization. Moreover, [^125^I]-iodomelatonin was shown to bind to mitochondrial membranes [209]. It will be of future importance to study directly the entrance, penetration and presence of melatonin in mitochondrial inner membranes.

Effects of the indoleamine on electron flux seem to have, at least, two aspects. Melatonin administration increased the activities of mitochondrial respiratory complexes I and IV in a time dependent manner in brain and liver [204,205,210]. However, these results were obtained in submitochondrial particles and, therefore, reflect activities of some more or less isolated proteins islets in the membrane, but not natural electron flux. What they do show is an improvement of electron transport capacity by melatonin. This is the more remarkable as these effects were also observed in aging and, especially, senescence-accelerated mice [211-213]. Some studies of this type were also accompanied by determinations of ATP [204,205,210]. With due caution, which is necessary because of the fact that a measured ATP concentration does not necessarily reflect ATP production rates, these results seem to indicate that also ATP formation is, in a sense, safeguarded by melatonin. If relevant, such effects should be also detectable at the level of the proton potential. In fact, processes perturbing the mitochondrial membrane potential such as calcium overload, either due to overexcitation, to protein misfolding or to damage by free radicals, are antagonized by melatonin. In cardiomyocytes, astrocytes and striatal neurons, melatonin prevented calcium overload [214,215], counteracted the collapse of the mitochondrial membrane potential induced by H_2_O_2 _[214], doxorubicin [216] or oxygen/glucose deprivation [215], and also inhibited the opening of the mitochondrial permeability transition pore (mtPTP), thereby rescuing cells from apoptosis. In addition to the antioxidant actions, melatonin directly diminished mtPTP currents, with an IC_50 _of 0.8 μM [215], a concentration that would require mitochondrial accumulation of melatonin, as discussed above.

Such findings require explanations exceeding the conventional antioxidant concept. In a recently proposed model [40,76], single-electron exchange reactions of melatonin are assumed to be the basis of interactions with the ETC, at low, quasi-catalytic concentrations. Under this perspective, radical scavenging by melatonin is not the principal, decisive property, but rather an indicator for melatonin's capability of undergoing single-electron transfer reactions. A cycle of electron donation to the ETC, e.g. at cytochrome c, followed by electron acceptance at N2 of complex I by the resulting cation radical was proposed. This cycle may reduce electron leakage at N2, with the cation radical as a potent competitor of O_2_. Such a cycle would enhance the net electron flux through ETC by diminishing electron leakage, thus safeguarding the proton potential and ATP synthesis [40,41,76]. In fact, reduction of electron leakage by melatonin was stated in neuroblastoma cells [217]. Moreover, similar properties were assumed for the melatonin metabolite AMK [40,41,76], which also easily undergoes single electron-transfer reactions and which is sufficiently amphiphilic, too [166,167]. Mitochondrial protection was, in fact, demonstrated also for AMK [203].

Indole antioxidants such as melatonin and their kynuric metabolites have multiple effects on oxygen and energy metabolism in improving, supporting and maintaining mitochondrial function and integrity [40]. In this context, the similarity of melatonin, its metabolites and other indolic as well as kynuric antioxidants to natural or synthetic electron and proton carriers such as ubiquinones and nitrones is remarkable and they all may be considered to act primarily as mitochondria-targeted bioenergetic agents [40,76,217]. By enabling, catalyzing and safeguarding single electron transfer reactions these mitochondrial antioxidants may enhance energy and oxygen metabolism efficacy and thereby act as very potent adaptogenic agents with profound neuroprotective activity [40,76,217]. Much like melatonin, nitrone and quinone compounds can prevent the mitochondrial toxicity of Aβ and thereby increase cellular viability and survival [28,91,120,217]. Since neuronal energy metabolism is strongly affected by Aβ [28,91,120,217], the preservation of mitochondrial activity may be one of the most important features shared by indole, nitrone and quinone antioxidant agents [28,91,120,217]. In aging and dementia, melatonin as well as any other mitochondrial metabolism modifier with a similar pharmacological profile may be able to restore brain energy supply, an activity that would distinguish these compounds from conventional antioxidant agents devoid of such neurotrophic effects. Catalytic antioxidants acting at the mitochondrial level would thereby allow for enhanced neuronal survival and synaptogenesis even under a severe amyloid burden [28,40,70,76,217]. Since mitochondria are a primary source and target of oxidative stress and damage, much of the neuroprotection seen after melatonin treatment in experimental models of AD, PD and HD may be somehow related to the specific effects of this indoleamine and its kynuramine metabolites in maintaining energy and oxygen metabolism of the organelles even under adverse conditions related to the neuropathology of these diseases.

### Melatonin and amyloid-β: antioxidant, antifibrillogenic and cytoskeletal effects

Several actions of melatonin have been described which antagonize the deleterious effects of Aβ. These actions concern different molecular processes, but may be interrelated; however, the possible connections require further investigation. One might classify the effects of melatonin as (i) antioxidant, including influences on mitochondrial metabolism, (ii) antifibrillogenic and (iii) cytoskeletal, including the suppression of protein hyperphosphorylation. Some of these actions were demonstrated at elevated, pharmacological concentrations, but any judgment of the relevance of such findings has to consider the relatively high rates of melatonin secretion into the CSF, uptake into the brain tissue and, presumably also, the metabolization to other protective compounds, such as the kynuramines AFMK and AMK [40,41], processes which are impaired during aging and in neurodegenerative diseases.

Attempts of using melatonin for antagonizing Aβ effects were based on the initial observation that the peptide induces oxidative stress, which leads to damage of mitochondrial DNA, formation of protein carbonyl, lipid peroxidation, changes in mitochondrial membrane structure, changes in respiration and breakdown of the mitochondrial membrane potential, induction of antioxidant enzymes and heat-shock proteins [3,106,218-224]. Notably, many of these findings were mitochondria-related. The pioneering work of Pappolla's research group first published compelling evidence for potent neuroprotection against the toxicity of Aβ in AD [218,225]. In fact, application of melatonin prevented the death of neuroblastoma cells exposed to Aβ peptide [91,142,225,226]. Similar results were obtained in astroglioma cells [227], findings of potential interest with regard to astrocyte-neuron interactions [228]. The indoleamine significantly reduced several features of apoptosis, like cellular shrinkage or formation of membrane blubs [225]. Additionally, lipid peroxidation in the cultured neuroblastoma cells was diminished, a finding first interpreted in terms of scavenging of free radicals generated by Aβ. However, and in accordance with our present point of view, the situation appears more complicated, since lipid peroxidation can also be a secondary consequence of mitochondrial dysfunction, and a support of mitochondrial integrity and electron flux should diminish the secondary formation of free radicals. It should also be noted that protection from Aβ-induced oxidative stress was achieved by the melatonin metabolite AFMK, too [164]. The relatively high concentrations required in this case may be seen on the background of the redox properties of AFMK, which preferentially undergoes two-electron transfer reactions and, therefore, is a less potent radical scavenger than its product AMK; this type of experiments should be repeated with AMK, which correspondingly disposes of a higher protective potential in mitochondria [cf. ref. [40]].

The second type of melatonin's antiamyloid actions concerns fibrillogenesis. Melatonin was shown by different techniques to inhibit the formation of amyloid fibrils, more efficiently than other, classical antioxidants [28,140,144,229]. Such effects were seen with both Aβ_1–40 _and Aβ_1–42 _peptides [229]. A structural analog of melatonin, indole-3-propionic acid, sharing the property of a good radical scavenger [230], had a similar or even higher antifibrillogenic activity [142,144]. Despite the similarities in redox chemistry, the effects on protein structure cannot be easily attributed to radical scavenging and are by far not understood. Notwithstanding, inhibition of amyloid plaque deposition by melatonin was also observed *in vivo*, using a transgenic mouse model [145,146]. Therefore, the antifibrillogenic actions are not just *in vitro *effects, although relatively high, pharmacological doses were required in the transgenics. However, despite the obvious histologically and behaviorally evident protection in these independent studies, antiamyloidogenic effects were not seen when the treatment was started in old transgenic mice, after 14 months of life [231]. In other words, after the disease has reached a certain severity, a substance like melatonin is no longer capable of efficiently antagonizing amyloid deposition and amyloid-dependent damage. However, nothing else should have been expected, after numerous amyloid plaques have been formed and neuronal damage has progressed. Consequently, one should see the value of melatonin mainly in its preventive potential rather than pinning unrealistic hopes on curative effects in later stages of disease. This does, however, not exclude symptomatic alleviations even in the progressed disease, concerning sleep, sedation, sundowning etc. (see below).

In the last years, the influence of lipoproteins on fibrillogenesis has received particular attention. Lipoproteins were found to interact with soluble Aβ, and levels of the free peptide may be crucial for parenchymal deposition [232]. However, the respective composition of lipoproteins including their content in cholesterol and apolipoprotein subtypes can modulate fibrillogenesis. Melatonin was shown to reverse the particularly profibrillogenic activity of apolipoprotein E4 and to antagonize the neurotoxic combinations of Aβ and apoE4 or apoE3 [140]. ApoE4, which aggravates Aβ effects, is also produced by astrocytes. A mutual potentiation between Aβ protein and apoE4 may, thus, be regarded as particular kind of astrocyte-neuron interactions in AD [228].

The third aspect, suppression of protein hyperphosphorylation and cytoskeletal disorganization, has largely been studied in experimental systems aiming to mimic by pharmacological means the changes which are typical of AD. Okadaic acid, a potent inhibitor of protein phosphatases 1 and 2A, not only induced cell death in two lines of neuroblastoma cells, but also mitochondrial dysfunction [82,86,87] and other characteristics of AD cytoskeletal changes (see above). Addition of melatonin prevented the okadaic acid-induced decline in cell viability and mitochondrial metabolic activity, attenuated lipid peroxidation and protected cytoskeletal integrity [86,87]. Similar data were obtained in neuroblastoma N2a cells, using calyculin A, another inhibitor of the same protein phosphatases. This study revealed an activation of GSK-3 (glycogen synthase kinase 3), a redox-controlled enzyme involved in various regulatory mechanisms of the cell [233]. Melatonin decreased not only oxidative stress and tau hyperphosphorylation, but also reversed GSK-3 activation, thereby showing that melatonin's actions exceeded its antioxidant effects, and also interfered with the phosphorylation system, especially stress kinases [233]. Tyrosine kinase (trk) receptors, representing other, particularly important elements of the phosphorylation system, and neurotrophins were also shown to be affected by oxidotoxins, including Aβ. In neuroblastoma cells, melatonin was capable of normalizing trk and neurotrophin expression [109]. In other experiments, tau hyperphosphorylation was induced by wortmannin [234] and isoproterenol [235]; again, melatonin was found to attenuate this process.

### Melatonin levels in Alzheimer's disease

Several studies show that melatonin levels are lower in AD patients compared to age-matched control subjects [236-241]. Decreased CSF melatonin levels observed in AD patients reflect a decrease in pineal melatonin production rather than a diluting effect of CSF. CSF melatonin levels decrease even in preclinical stages when the patients do not manifest any cognitive impairment (at Braak stages I-II), suggesting thereby that the reduction in CSF melatonin may be an early marker for the first stages of AD [242,243]. The reduction in nocturnal melatonin levels with the abolition of diurnal melatonin rhythmicity may be the consequence of dysfunction of noradrenergic regulation and depletion of the melatonin precursor 5-HT by increased MAO-A activity, as already seen in the earliest preclinical AD stages [242]. Alternately, changes in the pathways of light transmission, from physical properties of the dioptric apparatus to a defective retino-hypothalamic tract or SCN-pineal connections have been discussed as possible reasons of declines in melatonin amplitude and corresponding changes in the circadian system [244]. One should, however, be aware that light is inhibitory to the pineal [35,52], so that dysfunction in the transmission of light signals would not easily explain a decrease in melatonin. In any case, the changes in melatonin secretion could contribute to some frequent symptoms like sleep disruption, nightly restlessness and sundowning seen in AD patients [245]. Other reasons may be sought in an altered metabolism of AD patients, e.g., in relation to known genetic predispositions. The presence of apolipoprotein E-ε4/4, which is associated with enhanced Aβ toxicity and more rapid disease progression, also leads to considerably stronger declines in melatonin in the respective AD subpopulation than in patients with other apoE subtypes [238]. From this point of view, the relative melatonin deficiency may appear as a consequence rather than one of the causes of AD, although the loss in melatonin may aggravate the disease. Decreased nocturnal melatonin levels were also shown to correlate with the severity of mental impairment of demented patients [246].

### Sleep-wake and circadian rhythm abnormalities in AD patients

Despite the multifactorial etiology, the pronounced decline in nocturnal melatonin synthesis is common to AD patients. Therefore, the circadian system is impaired, and the circadian sleep-wake cycle is more strongly disturbed than in age-matched non-demented control subjects. The sleep-wake disturbances become more marked with progression of the disease. With the progressing neurodegeneration, the neuronal basis of the circadian system can be increasingly affected. Sleep-wake disturbances of elderly AD patients finally result from changes at different levels, such as reductions in the strength of environmental synchronizers or their perception, a lack of mental and physical activity, age- or disease-induced losses of functionality of the circadian clock. Cross-sectional studies have shown that sleep disturbances are associated with increased memory and cognitive impairment in AD patients [247].

AD patients with disturbed sleep-wake rhythms did not only exhibit reduced amounts of melatonin secreted, but also a higher degree of irregularities in the melatonin pattern, such as variations in phasing of the peak [239]. Therefore, the melatonin rhythm has not only lost signal strength in clock resetting, but also reliability as an internal synchronizing time cue. Loss or damage of neurons in the hypothalamic SCN and other parts of the circadian timing system may account for the circadian rhythm abnormalities seen in demented patients [240,248,249], especially as the number of neurons in the SCN of AD patients is reduced [248,250,251].

Clinical findings strongly argue in favor of disruption of the circadian timing system in AD, since numerous overt rhythms are disturbed, including body temperature and concentrations of other hormones such as glucocorticoids [252,253]. Circadian alterations, which are detectable at an advanced stage of AD, also concern phase relationships, such as the phase difference between the rest-activity and core body temperature cycles, the last one being significantly delayed [248,249]. Another criterion for a weakened circadian system may be seen in the possibility of improving rhythmicity in AD patients by well-timed light treatment [254]. In practical terms, this may be important as AD patients were found to be less exposed to environmental light than their age-matched controls [255], so that dysfunction of the SCN may be aggravated by low strength of the synchronizing signal light [256]. In other words, the AD patient is gradually deprived of the photic input and even more of the non-photic, darkness-related internal signal melatonin.

A chronobiological phenomenon in AD observed in conjunction with disturbances of the sleep-wake cycle is "sundowning ", symptoms appearing in the late afternoon or early evening, which include reduced ability to maintain attention to external stimuli, disorganized thinking and speech, a variety of motor disturbances including agitation, wandering and repetitious physical behaviours and perceptual and emotional disturbances [254,257]. A chronobiological approach with bright light, restricted time in bed and diurnal activity represents a therapeutic alternative for the management of sleep-wake disorders in AD patients [256]. Indeed bright light exposure in selected circadian phases markedly alleviated sundowning symptoms, such as wandering, agitation and delirium and improved sleep wave patterns in AD patients [258-260].

### Melatonin as a therapeutic agent for Alzheimer's disease

As outlined, melatonin acts at different levels relevant to the development and manifestation of AD. The antioxidant, mitochondrial and antiamyloidogenic effects may be seen as a possibility of interfering with the onset of the disease, although a balanced judgment requires due caution. While there can be no doubt that melatonin antagonizes Aβ toxicity and fibrillogenesis *in vitro*, at pharmacological levels also *in vivo *(see above), the beginning of treatment will be decisive [cf. ref. [231]]. One cannot expect a profound inhibition of disease progression once a patient is already in an advanced demented state, notwithstanding a very few case reports with anecdotal evidence of slight mental improvements [cf. refs. [28,261]]. Whether melatonin exerts a preventive effect, is a hope, but can be judged only after extensive epidemiologic studies. The possibility exists that melatonin is particularly useful in a subpopulation which is more susceptible to oxidative stress for reasons of genetic predispositions, such as defects in mitochondrial genes, apolipoprotein variants etc., and an epidemiologic evaluation will have to consider this complexity.

At least, melatonin has several obvious advantages over other comparable compounds, in particular, most other antioxidants. Because of its balanced amphiphilicity, it crosses the blood-brain barrier and enters any cellular compartment, including mitochondria [28,40,262].

The question whether melatonin has a causal value in preventing or treating AD, affecting disease initiation or progression of the neuropathology and the driving mechanisms, remains to be answered in future studies. Double-blind multicenter studies are urgently needed to further explore and investigate the potential and usefulness of melatonin as an antidementia drug. Its apparent usefulness in symptomatic treatment, concerning sleep, sundowning etc., even in a progressed state, further underlines the need for such decisive studies.

Melatonin as a sleep-promoting agent has been tried in a small non-homogenous group of elderly patients with primary insomnia (3 mg p.o. for 21 days) associated with dementia or depression. Seven out of ten dementia patients having sleep disorders treated with melatonin (3 mg p.o. at bed time) showed a significant decrease in sundowning and reduced variability of sleep onset time [263]. In another study, administration of 6 mg of melatonin to 10 individuals with mild cognitive impairment improved sleep, mood, and memory [264]. Similar observations were made by other groups, too. Seven AD patients who exhibited irregular sleep-wake cycles, treated with 6 mg for 4 weeks, showed a significantly reduced percentage of nighttime activity compared to a placebo group [49]. The efficacy of 3 mg melatonin/day at bedtime in improving the sleep and alleviating sundowning was shown in 11 elderly AD patients [265] and in 7 patients of another study [266]. Long-term administration of melatonin in the dose of 6–9 mg to 14 AD patients with sleep disorders and sundowning agitation for a period of 2–3 years improved sleep quality [267]. Sundowning, diagnosed clinically in all patients examined was no longer detectable in 12 patients. Another study on 45 AD patients with sleep disturbances, in which 6 mg of melatonin was given daily for 4 months, confirmed sleep improvement and suppression of sundowning [268]. Along with these ameliorations, which can already be seen as an important improvement, also with regard to the efforts of a caregiver, the evolution of cognitive alterations in melatonin receiving patients seemed to be halted in several individuals, as compared to AD patients not receiving melatonin.

The major findings were confirmed in a double-blind study, with regard to sleep-wake rhythmicity, cognitive and non-cognitive functions [269]. In a larger multicenter, randomized, placebo-controlled clinical trial, two dose formulations of oral melatonin were applied: 157 subjects with AD and nighttime sleep disturbance were randomly assigned to 1 of 3 treatment groups: (i) placebo, (ii) 2.5 mg slow-release melatonin, or (iii) 10 mg melatonin given daily for 2 months [270]. In this study, a statistical problem became apparent, since melatonin facilitated sleep in a certain number of individuals, but collectively the increase in nocturnal total sleep time and decreased wake after sleep onset, as determined on an actigraphic basis, were only apparent as trends in the melatonin-treated groups. On subjective measures, however, caregiver ratings of sleep quality showed significant improvement in the 2.5 mg sustained-release melatonin group relative to placebo [270]. Large interindividual differences between patients suffering from a neurodegenerative disease are not uncommon. It should be also taken into account that melatonin, though having some sedating and sleep latency-reducing properties, does not primarily act as a sleeping pill, but mainly as a chronobiotic. Since the circadian oscillator system is obviously affected in AD patients showing severe sleep disturbances, the efficacy of melatonin should be expected to also depend on disease progression.

The mechanisms that account for these therapeutic effects of melatonin in AD patients remain to be elucidated. Since the symptomatic actions become relatively rapidly apparent, they should be of mainly chronobiological nature. Melatonin treatment has been shown to promote mainly non-REM sleep in the elderly [56] and is found beneficial in AD by supporting restorative phases of sleep. Whether this includes in AD additional mechanisms known from non-demented elderly humans or animals, such as augmented secretion of GH [271] and neurotrophins [272], remains to be analyzed. The chronobiological aspect is underlined by a study on golden hamsters, in which melatonin was able to protect against the circadian changes produced by Aβ_25–35 _microinjection into the SCN [273]. From this point of view, changes in melatonin receptor density in AD – increases in arterial MT_1_[274] and decreases in hippocampal MT_2 _[275] – may be less important than a remaining responsiveness of the SCN, perhaps in conjunction with the sedating effects of melatonin based on downregulation of neuronal NO synthase and actions on the GABAergic system [276]. Regardless of the mechanistic details, all pertinent data unanimously direct to a sleep-promoting effect of melatonin in AD patients, as generally in elderly insomniacs [review: ref. [277]].

### Melatonin in Parkinson's disease

Parkinsonism, the other major neurodegenerative disease, is caused by a progressive loss of dopaminergic neurons in the substantia nigra. We do not refer to this disease with the intention of extensively reviewing here all its facets, but rather to outline some parallels with and differences to AD concerning the actions and applicability of melatonin. Oxidative stress was shown to play a major role in PD, too [278,279]. In post-mortem samples of the substantia nigra from PD patients, lipid peroxidation and oxidative modification of proteins and DNA were increased [26,280,281], whereas GSH was decreased [282]. A particular problem of vulnerability of the substantia nigra neurons is resulting from iron incrustations in the dopamine-derived melanins. The high levels of iron [280] are redox-active and generate hydroxyl radicals by the Fenton reaction. This situation appears to be aggravated by an enhanced rate of H_2_O_2 _formation, to which dopamine oxidation by MAO contributes [278].

Animal models of PD frequently use 6-hydroxydopamine (6-OHDA) to destroy the nigrostriatal pathway, or the neuronal oxidotoxin 1-methyl-4-phenyl-1,2,3,6-tetrahydropyridine (MPTP). Behavioral and motor deficits in rats or monkeys, such as akinesia, rigidity and tremor, reminiscent of those seen in PD patients [283], are commonly used to study the efficacy of therapeutic agents used in this disease.

MPTP administered to rats is mainly taken up by astrocytes and is metabolized into the 1-methyl-4-phenylpyridinium ion (MPP^+^). This cation is selectively taken up by dopaminergic neurons. Its actions are predominantly based on redox cycling involving redox-active enzymes [284] and, in particular, mitochondrial effects especially at complex I, thereby causing increased generation of free radicals, depletion of NAD and ATP and apoptosis [285,286]. In the 6-OHDA model, the neurotoxin acts selectively on nigrostriatal neurons because it is substrate of the reuptake transporter, and induces cell death by increased generation of free radicals due to autoxidation. In this context, one of the limits of l-DOPA medication may be noticed, namely, the iron-mediated hydroxylation of dopamine to 6-OHDA in the substantia nigra of PD patients [287].

Among the studies undertaken in animal models, some of them support the possible beneficial effects of melatonin in arresting the neurodegenerative changes, while others report adverse effects of melatonin in exacerbating motor deficits. In an MPTP model, melatonin counteracted the induced lipid peroxidation in striatum, hippocampal, and midbrain regions [288]. In a study using 6-OHDA, melatonin inhibited lipid peroxidation in cultured PC12 cells [289], effects that were associated with rises in antioxidant enzymes. Prevention of MPTP-induced dopaminergic cell death by melatonin was demonstrated by determining tyrosine hydroxylase levels and the number of normal DA cells [290].

The major MPP^+ ^effect, inhibition of complex I, leads to enhanced electron leakage, decrease of mitochondrial electron flux and ATP deficiency. Rises in complex I activity, as observed with melatonin [201-203,205], may antagonize this action and contribute to protection. Apart from such effects seen in submitochondrial particles, direct interactions of melatonin at N2 of complex I have been discussed [40,41,76]. In unilaterally 6-OHDA-injected, hemi-parkinsonian rats, protective effects by melatonin were also attributed to normalizations of complex I activity [291].

One should be aware that electron leakage at complex I causes secondary oxidative stress, by combination of the superoxide anions hereby formed with NO, to give peroxynitrite and radicals deriving from it (see above); in other words, a connection between mitochondrial dysfunction and the excitational vulnerability of the neuron becomes evident. In this regard, suppression of NO formation and scavenging of reactive nitrogen species by melatonin and its metabolite AMK [review: ref. [40]] should additionally support cell survival, along with other protective effects, such as upregulation of the antioxidant enzymes Cu,ZnSOD, MnSOD, GPx, which has been demonstrated in cultured dopaminergic cells, too [289].

While complex I inhibition is a plausible cause of neurodegeneration in the toxicological animal models, it would be of particular importance to know whether mitochondrial dysfunction is relevant in the PD patient. In fact, decreases in complex I activity were reported for mitochondria from platelets and in the substantia nigra of parkinsonian individuals [292-294]. However, recent investigations did not reveal any differences in complex I, II/III and IV activities in mitochondria from platelets [295], so that a genetically based dysfunction in electron transport is not evident. However, this does not entirely rule out striatal mitochondrial dysfunction in advanced stages of PD, because of an impairment by iron-mediated oxidative stress.

The pleiotropy of melatonin's antioxidant and otherwise protective effects is, on the one hand, a hindrance for relating cell survival to a particular, single mechanism in a given experimental situation, but, on the other hand, may give an impression of the powerful concerted actions of this indoleamine. Protection by melatonin was demonstrated in a variety of experimental PD models. [reviews: refs. [8,9,33]]. If studied in detail, the phenomenology of protection is complex, and melatonin may have acted on multiple targets, even though they may be partially interrelated. MPTP-induced stress was antagonized by melatonin at the levels of mitochondrial radical accumulation, mitochondrial DNA damage as well as breakdown of the proton potential [296]. As already outlined in the context of AD, cytoskeletal abnormalities are associated and, to a certain degree, caused by oxidative stress, but represent an own type of phenomenology, with additional regulatory mechanisms and additional sites of possible intervention. Lewy bodies, which are considered cytopathologic markers of parkinsonism, comprise abnormal arrangements of tubulin and microtubule-associated proteins, MAP1 and MAP2. Melatonin effectively promotes cytoskeletal rearrangements and was, thus, assumed to have a potential therapeutic value in the treatment of parkinsonism, and, perhaps, generally in dementias with Lewy bodies [297].

Recently, a possible melatonin-sensitive link between mitochondria, hyperphosphorylation and neuronal apoptosis became apparent, with general implications for mental deficits. In a study conducted in cerebellar granular neurons, melatonin did not only antagonize MPP^+^-induced cell death, but also activation of Cdk5 and cleavage of p35 to the hyperactivator p25 [298]. This protein kinase which has received its name for reasons of homology, but is unrelated to the cell cycle, seems to play an important role in neuronal function and plasticity. Dysregulation of Cdk5 and, in particular, rises in p25 have not only been found to occur in parkinsonism, but also in other neurodegenerative disorders including AD [299-301]. Moreover, inflammatory processes in the brain were shown to be associated with p25-dependent upregulation of Cdk5, along with tau hyperphosphorylation [302]. These findings are not only relevant in terms of neurodegeneration, but also with regard to cognitive processes in general. Cdk5 was shown to be required for associative learning, and its transient activation by p25 facilitates hippocampal long-term potentiation, in conjunction with increases in the density of synapses and dendritic spines [303-305]. However, this desirable effect on neuronal plasticity is turned into the opposite as soon as p25 formation and, thus, Cdk5 activation takes place for an extended period of time: a prolongued production of p25 led to synaptic and neuronal loss, impaired long-term potentiation and, consequenly, cognitive deficits [305,306]. Whether or not melatonin used at pharmacological concentrations in the MPP^+ ^study [298] influences p25 and Cdk5 activity indirectly via mitochondrial actions and/or directly by receptor-mediated signal transduction pathways, remains to be elucidated.

While all experiments on MTPT- or 6-OHDA-induced oxidative stress unanimously report protection by melatonin [288-290,307], the value of the pineal hormone may be judged entirely differently under systemic aspects. In rats treated with 6-OHDA or MPTP, pinealectomy or suppression of melatonin synthesis by bright light caused a remission of symptoms [308]. The view that melatonin may be unfavorable in the case of parkinsonism, was further supported by respective experiments using the (putative) melatonin receptor antagonists ML-23 and S-20928, which, again, improved motor functions and, in the case of ML-23, prevented 6-OHDA-induced mortality [309,310].

These findings show that antioxidative protection and even potentially beneficial mitochondrial effects do not suffice for judging the value of a drug under systemic aspects. The multiplicity of melatonin's actions, including the receptor-mediated ones, has to be a matter of responsible caution.

### Melatonin secretion in parkinsonism

Melatonin secretion patterns have been studied in patients suffering from PD. A phase advance of the nocturnal melatonin maximum was noted in L-DOPA-treated but not in untreated patients, as compared to control subjects [311-313]. Under medication with L-DOPA, daytime melatonin was additionally increased [313], a finding discussed in terms of an adaptive mechanism in response to the neurodegenerative process and possibly reflecting a neuroprotective property of melatonin [313].

In rats, fluctuations in serum melatonin levels were also related to variations in motor function and attributed to the interaction of monoamines with melatonin in the striatal complex [314]. Melatonin's inhibitory effect on motor activity has been suggested as one of the possible causes for the wearing-off episodes seen during drug treatment of parkinsonism. Electrical stimulation of internal globus pallidus inhibited an increase in daytime melatonin in PD patients as compared to healthy subjects [315]. Deep-brain stimulation of the internal globus pallidus had been shown to improve motor symptoms and complications in patients with Parkinson's disease [316].

### Melatonin's effects on sleep disturbances in Parkinson's disease

Studies undertaken in elderly insomniacs have convincingly demonstrated that melatonin can increase sleep efficiency and decrease nighttime activity [317,318]. Administration of melatonin in 5 mg/day for 1 week reduced the nocturnal wake time for about 20 minutes in eight patients with PD [319]. In a recent double-blind, placebo-controlled study on 40 subjects conducted over 10 weeks, Dowling et al. [320] noted that administration of a higher dose of melatonin, 50 mg/per day, increased actigraphically scored total nighttime sleep in PD patients, when compared with 5 mg or placebo-treated patients. Subjective reports of overall sleep disturbance improved significantly with 5 mg of melatonin compared to 50 mg or placebo [320]. This study may indicate that very high doses of melatonin can be tolerated in PD patients over a 10-week period as in healthy older adults. Nevertheless, the caveat from the melatonin-antagonist studies (see above) remains and should be taken serious.

### Melatonin in experimental models of Huntington's disease

Among neurodegenerative disorders, HD is the most clearly mitochondria-related disease. Primary cause is a mutation in the huntingtin gene, leading to an extended polyQ repeat, which causes protein misfolding and secondary effects hereof. Although huntingtin misfolding has multiple consequences, including some concerning iron metabolism [321], mitochondrial dysfunction is particularly fatal, in an excitation-dependent way. Under high calcium load, mitochondria carrying huntingtin with an extended polyQ domain are no longer able to cope with calcium uptake; as a result, complex II/III activity is impaired [322,323], the proton potential breaks down, and mtPTP-dependent cytochrome c release induces apoptosis [324,325]. Ca^2+ ^dependence explains the relationship to NMDA receptor-mediated excitation, and the selective vulnerability of frequently excited neurons carrying this receptor [326,327]. For these reasons, excitotoxicity by quinolinic acid, which also acts via the NMDA receptor, has been used as a model of HD [328-331]. Additionally, quinolinic acid has strong prooxidant properties when complexed with iron [332], a finding that is, however, uncertain with regard to its *in vivo *relevance. An alternate experimental model, using 3-nitropropionic acid as a blocker at complex II [329,333,334], acts primarily at the mitochondrial level, but is, in our experience, sometimes affected by the problem of ATP deficiency as the primary cause of cell death. One should clearly see the differences between the two models. Quinolinic acid acts upstream of the mutated protein, and most of the oxidative stress measured at low dosage of the drug sufficient for causing excitotoxicity may be regarded as side or secondary effects in the compromised cell. 3-Nitropropionic acid aims to mimic the mitochondrial blockade caused by misfolded huntingtin under calcium overload. In this case, oxidative stress can result from multiple sources and may include enhanced electron leakage.

Melatonin was shown to prevent quinolinic acid-induced lipid peroxidation in rat brain homogenates [335] and cell death in the rat hippocampus [336]. Since some effects of quinolinic acid differ from those of NMDA or glutamate and are obviously not mediated by its receptor, the question arose as to whether melatonin might protect mainly by antagonizing the NMDA receptor-dependent actions of the neurotoxin. Both quinolinic acid and NMDA induced lipid peroxidation in the rat hippocampus, but only damage by quinolinic acid was inhibited by melatonin; moreover, the action of melatonin was not inhibited by the MT_1_/MT_2 _blocker luzindole [337]. Therefore, one should conclude that, at least, the induction of lipid peroxidation is not mediated via the NMDA receptor, nor the antagonizing effect of melatonin via its membrane receptors. Extensive lipid peroxidation after administration of quinolinic acid was not only seen in hippocampal, but also striatal and globus pallidum regions, again antagonized by melatonin, which additionally attenuated neurobehavioral signs associated with the neurotoxin [338]. In brain tissue culture, melatonin antagonized the prooxidant effects of high doses of quinolinic acid, which strongly exceed the concentrations required for excitotoxicity [339]. Lipid peroxidation induced by 3-nitropropionic acid in synaptosomes of rat striatal and cortical regions were attenuated by melatonin [340]. Collectively, these results demonstrate the antioxidant capacity of melatonin, but the relevance for HD may greatly depend on the validity of the animal models for fully describing the situation in the disease. Melatonin's undoubtedly existing antiexcitotoxic properties are not clearly apparent in studies focusing on lipid peroxidation.

## Conclusion

The most striking feature of melatonin is its pleiotropy, with regard to both target cells and mechanisms. Any consideration of the possible value of melatonin has to take this into account and to weigh advantages and eventual disadvantages of effects exerted at the various levels of action. A balanced and responsible view will only be achieved if the meaning of the multiplicity of actions is clearly seen and distinctions are made between the various experimental systems and the relevance of their outcome relative to the situation in a patient.

Without any doubt, melatonin is one of the most powerful antioxidants acting at various levels, from direct radical scavenging and enzymatic regulation of oxidant formation to mitochondrial radical avoidance [40]. Additionally, indirect antioxidant effects are based on support of appropriate circadian phasing and antiexcitatory or antiexcitotoxic actions [40,76]. On this background, it is not surprising that melatonin has proved to be protective in numerous experimental systems in which oxidative stress is generated directly or indirectly, in cell and tissue cultures, but also in animals. The prevention of apoptotic or necrotic cell death can be partially attributed to this property, but additional mitochondrial effects concerning the support of electron flux, proton potential, ATP synthesis and direct inhibition of the mtPTP [215] can be decisive. In a neuron which is more vulnerable to overexcitation for genetic reasons, antiexcitatory effects of melatonin may already be sufficient for rescuing the cell. One has to distinguish between these possibilities by appropriate experimental approaches. In any of these cases, in which either antioxidant – in the broadest sense – antiexcitatory or antiapoptotic effects are prevailing, melatonin will be found to be protective.

Nevertheless, one should not forget to what extent the model systems represent artificial situations, which can only partially portray the disease of a patient, and which are frequently based on powerful pharmacological or toxicological means. Consequently, doses of melatonin required are frequently in an upper pharmacological range, too, setting limits to the judgment on melatonin's value. With all due reserve, one can, however, state that the application of melatonin is still a source of hopes for possibilities of intervention, also because melatonin is usually remarkably well tolerated by the treated individual, contrary to many other medications. Long-term administration of oral melatonin of 30 or 60 mg per day in a slow-release formulation was surprisingly unproblematic and safe in ALS patients [341]. In a more recent study on 31 ALS patients, even 300 mg of rectally administered melatonin was tolerated without problems for 2 years [342]. In numerous other studies mentioned in this review, lower doses were also unproblematic.

Caution seems due at the present state of our knowledge in the case of PD. At least in rat models, suppression of membrane receptor-mediated melatonin effects alleviated symptoms induced by 6-OHDA or MPP^+ ^[308-310]. This should be taken as a caveat with regard to eventual unfavorable effects on disease progression. On the other hand, it became obvious that melatonin is promoting sleep efficiency also in PD patients. How risk and benefit have to be weighed in humans suffering from this disease remains to be elucidated.

Contrary to this, the balance seems to be largely in favor of melatonin in the case of AD. Apart from the positive effects in experimental systems concerning antagonism of oxidative stress, fibrillogenesis and tangle formation, the sleep-promoting effects – even if not demonstrable in all individuals – and the suppression of sundowning are important results justifying the use melatonin. Mild cognitive improvements should also be welcome. The problem in AD remains to which extent melatonin may be effective in retarding disease progression. One should not expect too much in an advanced state. Nevertheless, the preventive potential of melatonin deserves attention and continued investigation. Even from a cautious and realistic, perhaps even sceptical point of view, the findings obtained to date should be taken as a good reason for planning further multicenter trials, in which, however, the collectives of patients have to be large enough for distinguishing between different stages of disease progression. Whether or not melatonin may have a preventive potential might become clear in subpopulations of high-risk individuals, e.g. those with pertinent familial history or carrying unfavorable apolipoprotein variants.

With regard to prevention, melatonin should also be seen in the general context of aging. In the past, this has been a matter of controversy, but mainly for methodological reasons. Recent studies show that age-dependent patterns of gene expression can be reverted to a more juvenile state in the mouse CNS [343]. Life extension with melatonin is possible in model animals, but melatonin's value is not only a matter of life-span, but also of health during aging, and pertinent observations have, in fact, been made in mammals [262].
